# StaticPigDetv2: Performance Improvement of Unseen Pig Monitoring Environment Using Depth-Based Background and Facility Information

**DOI:** 10.3390/s26020621

**Published:** 2026-01-16

**Authors:** Seungwook Son, Munki Park, Sejun Lee, Jongwoong Seo, Seunghyun Yu, Daihee Park, Yongwha Chung

**Affiliations:** 1 Info Valley Korea Co., Ltd., Anyang 14067, Republic of Korea; sso7199@invako.kr (S.S.); lllss22@invako.kr (S.L.); 2 Department of Computer Convergence Software, Korea University, Sejong 30019, Republic of Korea; mkp990302@korea.ac.kr (M.P.); seojongwoong@korea.ac.kr (J.S.); tidlsld44@korea.ac.kr (S.Y.); dhpark@korea.ac.kr (D.P.)

**Keywords:** pig detection, deep learning, video monitoring, static camera, occlusion

## Abstract

**Highlights:**

The three graphs present overall performance improvements in accuracy and latency across different YOLO models. The red lines indicate the baseline models, while the blue lines indicate the proposed models. The black lines represent the performance changes for each model, highlighting the improvement from the baseline to the proposed method.

**What are the main findings?**

**What is the implication of the main finding?**

- With YOLOv8, YOLOv10, and YOLOv12 nano models, the proposed method improves the baseline accuracy, while slightly increasing the execution time due to the fixed preprocessing operations, by exploiting the background and facility information.
- With YOLOv8, YOLOv10, and YOLOv12 small, medium, and large models, however, the proposed method improves both the baseline accuracy and execution time because the portion of the fixed preprocessing operations is relatively decreased with bigger models.

**Abstract:**

Standard Deep Learning-based detectors generally face a trade-off between accuracy and latency, as well as a significant performance degradation when applied to unseen environments. To address these challenges, this study proposes a method that enhances both accuracy and latency by leveraging the static characteristics of fixed-camera pig pen monitoring. Specifically, we utilize background and infrastructure information obtained through a one-time preprocessing step upon camera installation. By integrating this information, we introduce three distinct modules, Background-suppressed Image Generator (BIG), Facility Image Generator (FIG), and Background Suppression Integration (BSI), that improve detection accuracy and operational efficiency without the need for model retraining. BIG creates background-suppressed images that integrate foreground and background information. FIG creates facility mask images that can be used to identify pigs that are occluded by facilities, enabling more efficient learning in unseen environments. BSI leverages both the input image and the background-suppressed image generated by BIG, feeding them into a 3D convolution layer for efficient feature fusion. This difference-aware fusion helps the model focus on foreground information and gradually reduce the domain gap. After training on the German pig dataset and testing on the unseen Korean Hadong pig dataset, the proposed method could improve AP_50_ accuracy (from 75% to 86%) and Jetson Orin Nano latency (from 67 ms to 41 ms) compared to the baseline model YOLOV12m.

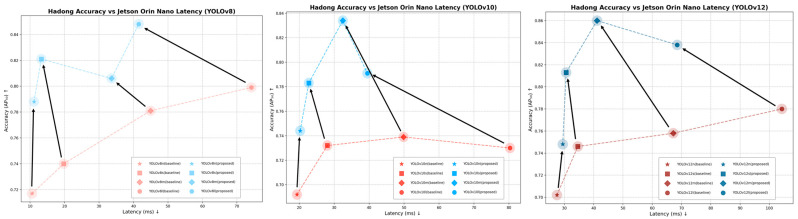

## 1. Introduction

Pork consumption is projected to grow at an average annual rate of approximately 1.2% over 2018–2027 [1]. As production scales, management that relies solely on human labor faces inherent limitations in cost, consistency, and coverage, which motivates automated pig monitoring systems [2,3,4]. Computer vision has therefore been widely adopted for monitoring tasks such as pig detection, counting, tracking, posture recognition, and behavior analysis in commercial barns [5,6,7,8,9]. By training object detectors to localize pigs in images, the positions of pigs can be estimated and subsequently used as inputs for downstream management functions within each pen (e.g., counting, tracking, and activity analysis) [10,11,12,13,14,15,16].

Despite recent progress, deep learning-based detectors often perform well only when the deployment environment is similar to the training environment, where the model has already learned the visual patterns of that specific scene which is “Seen Environment”. When the trained model is deployed in an environment with different backgrounds, facility structures, occlusions, illumination, or camera viewpoints not included in training dataset, performance can degrade substantially due to domain shift [17,18], which is “Unseen Environment”. Reducing the gap between seen and unseen environments is therefore crucial for achieving stable and reliable pig detection in practical monitoring systems.

**Figure 1:**
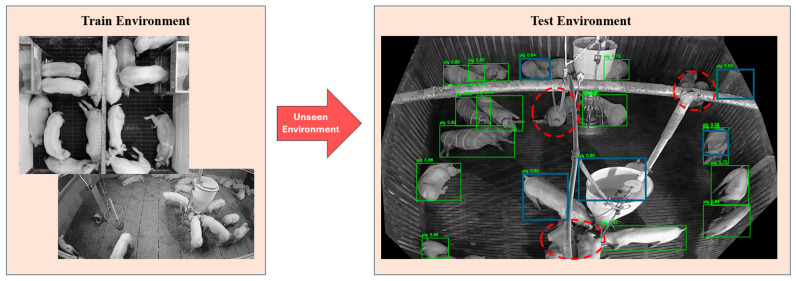
Detection errors in unseen pig pen test environment trained in a completely different environment. Green boxes show correctly detected cases. Red dotted circles show missed detection errors and blue rectangles show falsely detected errors.

Figure 1 illustrates typical failure cases in a commercial pig pen environment. The detector is trained using images collected from a different barn with different flooring, facility occlusions, and viewpoints, and the test environment is unseen during training. This domain discrepancy produces diverse detection errors, including missed detections under pipe occlusion and false positives in which facility components (e.g., walls) are misclassified as pigs. In pig production facilities, such domain shift is particularly critical because farm layouts, facility designs, and lighting conditions vary considerably, and pigs are frequently occluded by fences, feeders, and other equipment [19]. Conventional detectors may inadvertently learn spurious correlations between pig appearance and environment-specific background cues, which can lead to overfitting to a particular facility and poor generalization to new barns or camera viewpoints. Consequently, detectors that are accurate in the training environment can still miss pigs or produce unstable outputs in unseen facilities [17,18].

For continuous 24/7 monitoring in commercial barns, pig detection models must operate in real time on cost-effective embedded hardware, where computational resources and power budgets are limited [14,16]. Accordingly, we adopt YOLO-based detectors as a baseline due to their favorable accuracy–latency trade-off and practical deployment maturity for real-time applications [20]. However, because the structural layout and background of each pig pen differ substantially, conventional models often experience a sharp drop in accuracy in unseen environments [17,18]. While domain adaptation and self-training can mitigate this issue, they typically require additional environment-specific tuning or training procedures for each new target facility [18]. Unlike conventional domain adaptation or domain generalization methods that enforce domain-invariant representations through additional training objectives, this study adopts a deployment-driven generalization strategy by explicitly removing domain-specific nuisance factors at the input and feature fusion stages.

In this study, we propose StaticPigDetv2, a pig detection framework designed to reduce the gap between seen and unseen environments by explicitly modeling background and facility information [21]. StaticPigDetv2 introduces three modules: Background-suppressed Image Generator (BIG), Facility Image Generator (FIG), and Background Suppression Integration (BSI). BIG uses depth-based background estimation together with Depth Anything V2 [22] and Inpaint Anything [23] to generate background-suppressed images that emphasize foreground pig cues while suppressing environment-specific details. FIG models facility structures so that pigs occluded by equipment can still be inferred using contextual information from the surrounding scene. Finally, BSI fuses the complementary representations acquired from BIG and FIG to train the detector with explicit foreground and facility cues, thereby encouraging robust detection under facility occlusion and across diverse barn environments.

We use an inference-time input preprocessing strategy that improves both accuracy and latency by leveraging recent Transformer-based vision models—Depth Anything V2 [22] and Inpaint Anything [23]—to extract background and infrastructure information from the monitored pig pen. Although Transformer-based models are usually computationally demanding, we consider a deployment scenario in which they are executed only once during the initial static-view camera installation. The static information obtained from this one-time execution is then reused to enhance detection robustness during real-time inference, without imposing additional computational burdens on the embedded detection pipeline.

The remainder of this paper is organized as follows. Section 2 reviews related work; Section 3 describes StaticPigDetv2 and the BIG, FIG, and BSI modules; Section 4 presents experimental results; and Section 5 concludes the paper and suggests directions for future work.

The contributions are as follows:

1. We propose a method that extracts background and facility information without additional training per unseen environment as additional training for each seen environment is not practical.
2. We propose a feature fusion method that utilizes 3D convolution to effectively fuse the extracted information, improving both detection accuracy and latency. The overall performance gains observed across multiple YOLO models demonstrate the effectiveness of the proposed approach.

## 2. Related Works

The primary objective of this study is to address the performance degradation that occurs when deep learning-based detectors are deployed in unseen pig farm environments. Research has evolved from automated counting [10] and part detection [24] to sophisticated posture analysis [25,26,27,28,29,30,31,32]. While these advancements are significant, a persistent challenge remains achieving high detection accuracy while maintaining the low latency required for commercial viability [14,33]. Current state-of-the-art architectures, such as YOLOv10 [34] and YOLOv12 [35], offer impressive real-time performance but still struggle with the domain shift caused by varying farm layouts and facilities [17,36].

In large-scale smart farming, system latency is a critical constraint [14,33]. Monitoring is typically conducted via overhead surveillance streams [12,37], requiring lightweight models [38,39] or ensemble-based post-processing [40] to ensure reliable results on embedded boards [14,16]. To provide long-term data, tracking algorithms [12,13,15,16,41] follow individual pigs to detect health anomalies [42,43]. Recent trends emphasize the use of spatiotemporal information and graph convolutional networks to analyze complex social behaviors and feeding patterns [44,45,46].

Recent years have seen a transition toward more granular detection. Center clustering networks [36] and PDC-YOLO [47] have been developed to handle heavy occlusion in counting tasks. Furthermore, 3D point cloud segmentation [48] and dual-attention feature pyramid networks [49] have improved instance segmentation in crowded group-housed pens. Posture recognition now extends beyond basic lying or standing [8,25] to include engagement with enrichment objects [50], drinking behavior [29], and interaction recognition [51]. Systems like DigiPig [52] and hybrid YOLO-EfficientNet workflows [53] allow for 24/7 monitoring of physico-temporal activities [54].

Environmental challenges, such as dust-induced blur, often necessitate specialized denoising [19]. While traditionally used for motion detection via difference-imaging [15], background data also helps identify static pen facilities. Distinguishing subjects from infrastructure is essential for detecting specific targets like sows [55] or pig faces [56]. Our proposed Background-suppressed Image Generator (BIG) and Facility Image Generator (FIG) formalize this by utilizing one-time preprocessing to filter static noise.

Standard detectors frequently fail in unseen environments due to shifts in flooring, lighting, and internal facilities [17,36]. While systems may perform well in specific zones like hallways [16], environmental distribution shifts between training and deployment sites often lead to failure [39,43]. Recent research has explored self-training with augmented target dataset to mitigate this domain shift [18]. However, applying heavy foundation models like Depth Anything V2 [22] or Inpaint Anything [23] is often impractical for real-time monitoring due to computational limits. This necessitates efficient fusion methods, such as our Background Suppression Integration (BSI), which bridges the domain gap without requiring model retraining.

The works that are similar to the proposed works are StaticPigDet [21] and DOG [17]. In StaticPigDet [21], background and facility information were exploited to improve detection accuracy, particularly for pig objects occluded by facilities. However, StaticPigDet [21] still has several limitations related to unseen environments and reliance on surveillance video. Its experiments were conducted on datasets collected from a single environment, which may reduce detection accuracy under unseen conditions such as different flooring, lighting, or new facility structures. In addition, the background and facility images require long surveillance videos captured by static cameras, which may not be available for all farms.

In DOG [17], pig object detection was improved in unseen pig farm environments. By leveraging foundation models [22,23], foreground and background information were extracted to identify regions of interest (RoIs) [50], and this information was used as an additional input to improve object detection accuracy. However, this approach does not explicitly consider pigs occluded by facilities, which is a frequent scenario. Also, accuracy was only increased, but process speed decreased due to preprocessing.

While several studies consider unseen environments and some make use of background images; to the best of our knowledge, no prior work jointly addresses both factors. Recent studies that address unseen environments and utilize background images are summarized in Table 1.

## 3. Proposed Method

**Figure 2:**
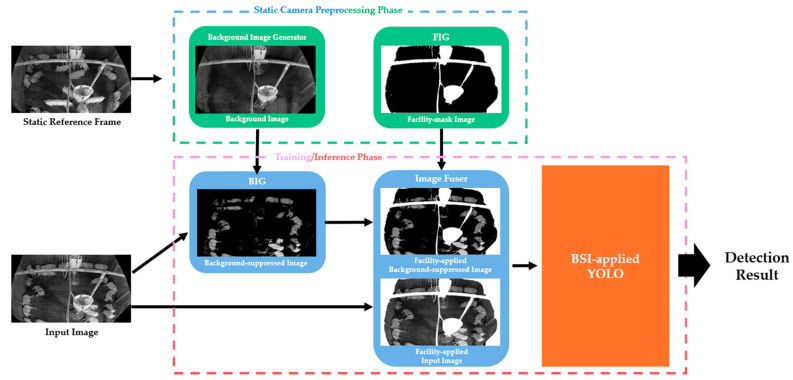
Overview of the proposed method StaticPigDetv2.

Differences in the object detection environment under unseen conditions lead to detection errors, because the new environment is not represented in the training dataset. To mitigate this issue, this study proposes a method that addresses accuracy degradation caused by environmental differences using image processing techniques. We propose BIG, FIG, and BSI modules to utilize foreground, background, and facility information, respectively. Background image and facility information is created once per static environment, thus naming it the Static Camera Preprocessing Phase. The spatial locations of each type of information are used to differentiate the various components of the image and to fuse them accordingly. The overview structure of the proposed method is as shown as Figure 2.

First, a background image and facility image are created from a static reference frame using Background Image Generator and FIG during the Static Camera Preprocessing Phase. Then, background-suppressed image is created by using the input image and generated background image. Then, facility mask information acquired from FIG is applied to both the input image and the background-suppressed image to create facility-applied background-suppressed image and facility-applied input image. Finally, BSI-applied YOLO is trained and inferenced using two inputs: facility-applied background-suppressed image and facility-applied input image.

### 3.1. Background-Suppressed Image Generator

**Figure 3:**
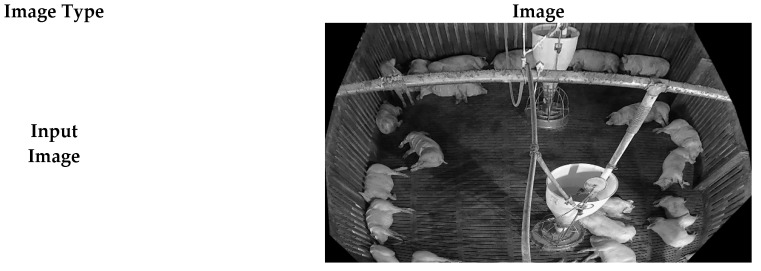

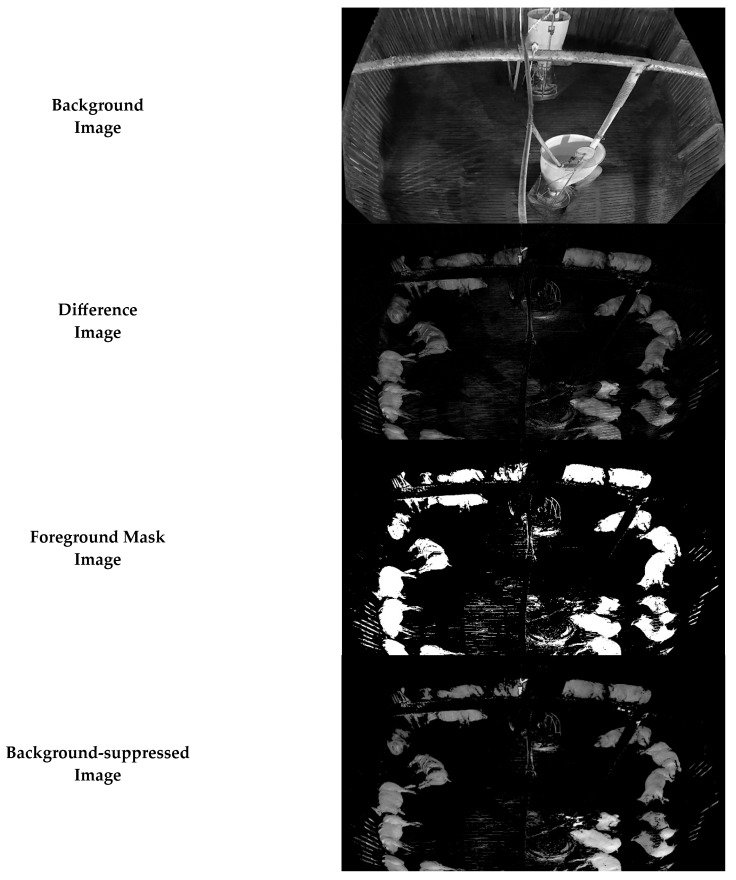
Example images of each step processed in BIG.

In unseen environments, background regions that are absent during the training phase often cause detection errors because they are not represented in the training dataset. The proposed BIG module generates foreground-emphasized images in which the background is suppressed, allowing the network to focus on foreground features during training. Each step of this generation process is illustrated in Figure 3.

First, in the Background Image Generator during the Static Camera Preprocessing Phase, Inpaint Anything [23] is applied to a static reference frame to remove the detected pig foreground and to synthesize an appropriate background. The process is executed once per static environment rather than for every input image as this procedure is performed during the Static Camera Preprocessing Phase. Next, the generated background image is subtracted from the input image to produce a difference image, and the absolute value function is applied to ensure non-negative pixel values. In this difference image, pixels corresponding to background regions have values close to zero, whereas pixels at foreground locations have higher values. Otsu’s thresholding is then applied to the difference image to generate a foreground mask, in which pixels with higher values (foreground pixels) are labeled as foreground (e.g., value = 255) and near-zero pixels (background pixels) are labeled as background. Finally, background-suppressed images are created by applying the foreground mask to the input image: pixel values at foreground locations (mask value ≥ 128) are preserved to maintain the original texture of the pig objects, while pixel locations identified as background (mask value < 128) are set to 0. The overall process is summarized in Algorithm 1.
**Algorithm 1**. Background-suppressed Image Generator**Input:**
 $\mathrm{Image}:\;\mathrm{Input}\;\mathrm{Image}\;f_{Input}$$,\;\mathrm{Background}\;\mathrm{Image}\;f_{Background}$
**Output:**
  Background-suppressed Image $f_{output}$$f_{Difference}$$\;=\;abs(f_{Input}-\;f_{Background})\;$ $f_{ForegroundMask}$ $=\;\mathrm{Apply}\;\mathrm{Otsu}\;\mathrm{thresholding}\;\mathrm{on}\;f_{Difference}$ **If** $f_{ForegroundMask}\left[y,x\right]$ > 128 then
 $f_{output}[y,x]$$\;=\;f_{Input}[y,x]$ **Else**
 $f_{output}[y,x]$ = 0
$\mathrm{Return}\;f_{output}[y,x]$


### 3.2. Facility-Mask Image Generator

**Figure 4:**
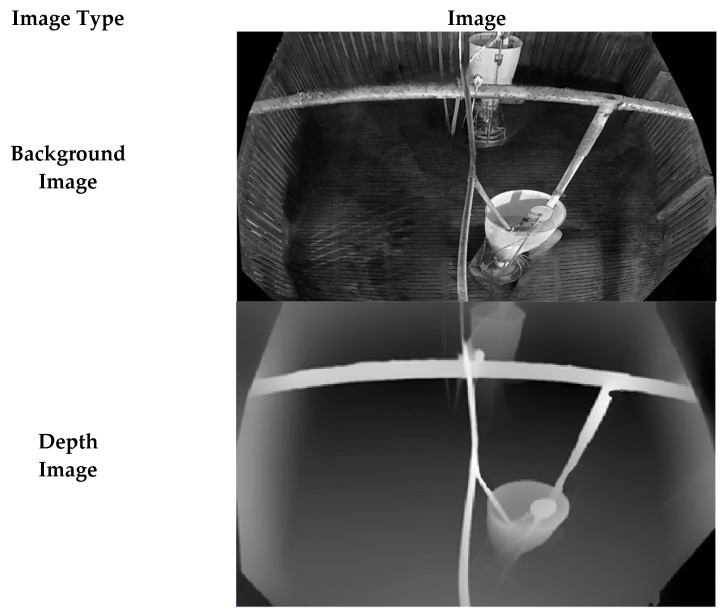

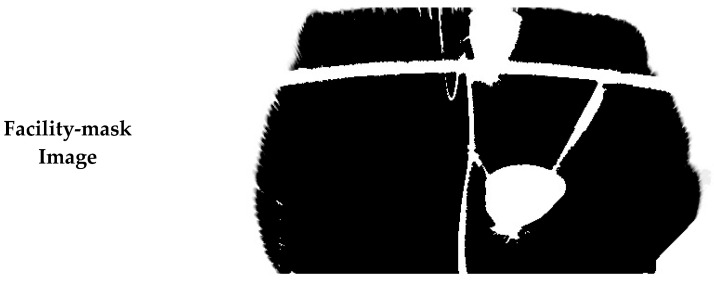
Example images of each step processed in FIG.

Many detection errors arise when pig objects are partially or fully occluded by facilities in the pig farm. A key issue is that the heterogeneous visual characteristics of these facilities, when blocking pigs, lead to missed detections. Therefore, if facility locations can be identified, this information can be leveraged to improve detection accuracy. Each step of the FIG process is illustrated in Figure 4.

First, during the Static Camera Preprocessing Phase, Depth Anything [22] is used to generate a depth image, $f_{Depth}$, from the background image, $f_{\mathrm{Background}}$, where pixel values represent distance from the camera, thereby making facility structures more distinguishable. Higher pixel values indicate regions closer to the camera, whereas lower values correspond to regions closer to the pen background. Next, Otsu’s thresholding is applied to $f_{Depth}$ to generate a facility mask image, $f_{FacilityMask}$, which separates regions at different depth levels and enables more precise identification of facility areas. In $f_{FacilityMask}$, pixels with values greater than 128 are treated as foreground and set to 255, whereas the remaining pixels are set to 0.

Finally, in the Image Fuser, the identified facility regions are assigned a pixel value of 255 in both the input image and the background-suppressed image so that they are visually masked and facility textures are equalized. Because this procedure is performed during the Static Camera Preprocessing Phase, it is executed once per static environment rather than for every input image. (Algorithm 2).
**Algorithm 2**. Facility-mask Image Generator**Input:**
  Image: Background Image $f_{Background}$
**Output:**
 Facility-mask Image $f_{output}$$f_{Depth}$$\;=\;\mathrm{Apply}\;\mathrm{DepthAnything}\;\mathrm{on}\;f_{Background}$ $f_{FacilityMask}$$\;=\;\mathrm{Apply}\;\mathrm{Otsu}\;\mathrm{thresholding}\;\mathrm{on}\;f_{Depth}$ **If** $f_{FacilityMask}\left[y,x\right]$ > 128 then 
 $f_{output}[y,x]$ = 255

**Else**

 $f_{output}[y,x]$ = 0

$\mathrm{Return}\;f_{output}[y,x]$


### 3.3. Background Suppression Integration

**Figure 5:**
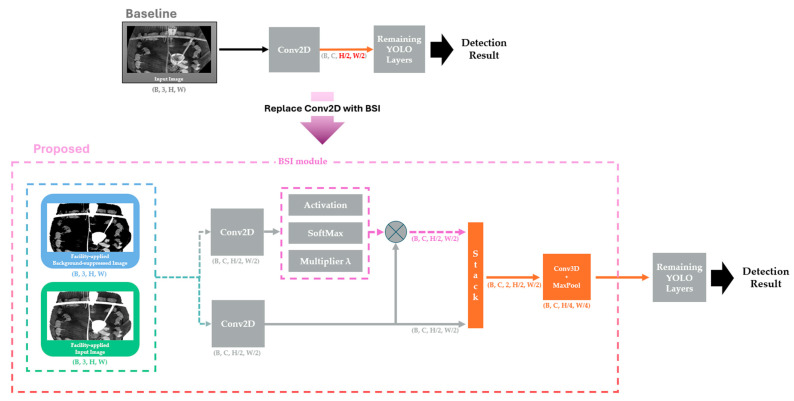
Overview of the baseline YOLO architecture with the proposed Background-Suppressed Integration (BSI). The diagram illustrates how the proposed BSI differs from the baseline method. As highlighted in red, the proposed method reduces the input height and width by a factor of four, whereas the baseline reduces them by a factor of two, thereby improving inference latency. In addition, the proposed BSI uses two image inputs rather than the single-image input used in the baseline method, thereby improving accuracy.

Figure 5 presents an overview of BSI architecture. To fully exploit the background-suppressed image, we extend the baseline single-input detector to a dual-input architecture. The model takes two image tensors as input, each with shape $\left(B,3,H,W\right)$, where B, H, and W denote the batch size, height, and width, respectively. These two inputs capture different aspects of the scene, enabling the network to learn complementary representations from the facility-applied background-suppressed image and the facility-applied raw input image.

Each input is first processed by a 2D convolution layer with stride 2, reducing the spatial resolution by half and producing two feature maps of shape $\left(B,C,H/2,W/2\right)$, where C is the number of channels. For the background-suppressed branch, an activation function followed by a SoftMax operation is applied to obtain an attention-like feature map. This feature map is then scaled by a multiplier λ to increase its influence during fusion. The scaled map is used to guide feature selection by modulating the feature map from the input-image branch through element-wise multiplication, thereby emphasizing informative regions and attenuating irrelevant ones.

To further enhance feature interactions, we incorporate a 3D convolution layer to fuse the two feature maps more effectively. Specifically, the two 4D feature maps $\left(B,C,H/2,W/2\right)$ are stacked along a new depth dimension to form a 5D tensor $\left(B,C,2,H/2,W/2\right)$, which is then passed to a 3D convolution layer whose channel size matches that of the preceding 2D convolution in BSI. This operation produces a fused representation with an additional depth dimension. The output is subsequently reduced back to a standard 4D feature map $\left(B,C,H/4,W/4\right)$ using max pooling, preserving the fused information for downstream 2D convolution layers. In BSI, stride 2 is applied in the spatial dimensions to mitigate the computational overhead introduced by the 3D convolution while retaining the combined information from both input streams.

## 4. Experimental Results

The study was conducted on an Intel Core i7-7700K @ 4.20 GHz processor (Intel Corporation, Santa Clara, CA, USA) and a GeForce RTX 3060 GPU (NVIDIA Corporation, Santa Clara, CA, USA). The operating system was Ubuntu 22.04 LTS (Canonical Ltd., London, UK), and the deep learning models were implemented in PyTorch 2.0.1 (Meta Platforms, Inc., Menlo Park, CA, USA) with CUDA 11.7 (NVIDIA Corporation, Santa Clara, CA, USA). For embedded-board evaluation, inference latency were additionally measured on an NVIDIA Jetson Orin Nano Developer Kit (NVIDIA Corporation, Santa Clara, CA, USA) running JetPack 6.2 (Linux for Tegra, Ubuntu-based). The same trained weights were deployed to the Jetson device, and all runtime measurements were collected with batch size 1 after an initial warmup.

**Figure 6:**
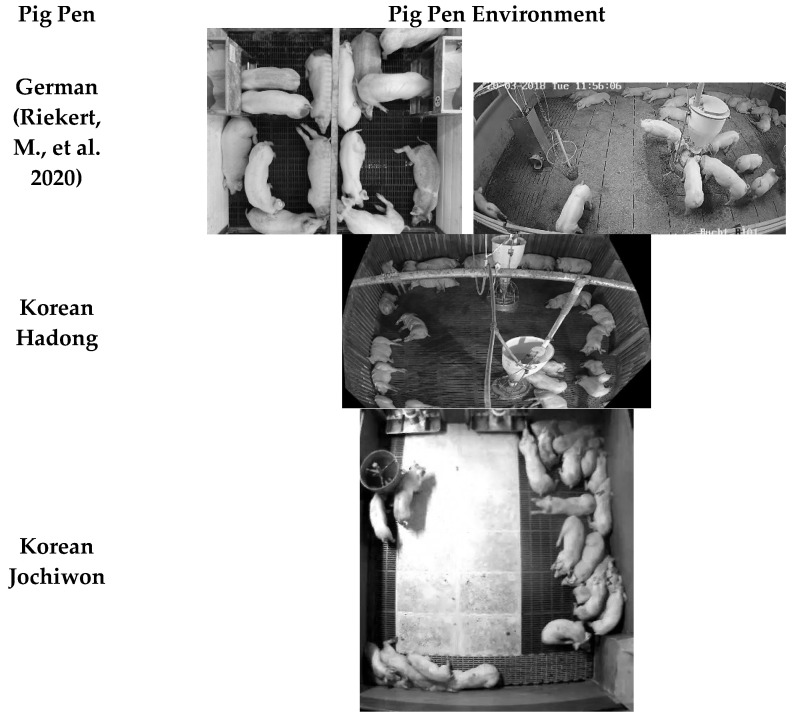
Visual representation of different pig pen environments [27].

Figure 6 shows the example of each pig dataset. The training dataset was taken from the German pig dataset [27], using 788 images for training, named “German”. The main experiments were conducted on a test dataset collected from a pig pen, located in Hadong-gun, Gyeongsangnam-do, Korea, which contained 200 images, named “Hadong”. The Hadong dataset was used as characteristics of large feeding facility and ceiling pipes occlusion as well as a camera deployed at 45 degrees tilted-view. Additional ablation studies to show robustness for unseen environment was conducted in a pig pen located in Jochiwon-eup, Sejong-si, Korea with total of 210 images, named “Jochiwon”. To collect this dataset, a camera was deployed on the ceiling of the Jochiwon pig pen, approximately 3 m above the floor showing top-view. The Jochiwon dataset has characteristics where many pigs tend to occlude one another in a group; thus, multiple pigs are detected as one pig. In terms of labels, only pigs that show more than a 20% visible region were labeled in both the training and test datasets. A perspective transformation image processing technique was applied to regularize the size of pig objects for the training and test dataset.

All models were trained with an input resolution of 640 × 640 for 100 epochs, and a fixed batch size of 8 and SiLU was used for activation function. For all YOLOv8/10/12 variants, BSI replaced the first convolution layer in the backbone, keeping the remainder of the architecture unchanged. The detector architectures used for training and testing were YOLOv8 [20], YOLOv10 [34], and YOLOv12 [35], and for each model, the n, s, m, and l variants were evaluated. We used the default training setting regarding optimizer, initial learning rate, learning rate schedule, weight decay, momentum, and warmup of the official YOLOv8/10/12 implementations.

We evaluated all models using Precision, Recall, AP_50_, and F1-Score, and report inference latency in ms measured on the RTX 3060 and Jetson Orin Nano with batch size 1. Specifically, AP_50_ denotes the average precision at an IoU threshold of 0.5, where IoU is defined as Equation (1).

(1) $$\begin{array}{cccc} & IoU(B,G)=\frac{|B\cap G|}{|B\cup G|}, & & \end{array}$$

In Equation (1), B is the predicted bounding box, G is the ground-truth bounding box, ∣B∩G∣ is the intersection area between B and G, and ∣B∪G∣ is the union area between B and G. A detection is considered a true positive when IoU≥0.5 with the correct class (one-to-one matching). Precision and Recall are computed as $Precision=\;\frac{TP}{TP+FP}$ and $Recall=\frac{TP}{TP+FN}$, where TP is the number of true positives, FP is the number of false positives, and FN is the number of false negatives.

AP_50_ is computed as the area under the precision–recall curve at IoU = 0.5, which is given in Equation (2).

(2) $$\begin{array}{cccc} & {AP}_{50}=\int_{0}^{1}p(r)\;dr\approx \sum_{k}(r_{k}-r_{k-1})\;p_{\mathrm{interp}}(r_{k}), & & \end{array}$$

In Equation (2), p(r) denotes precision as a function of recall r, r∈[0,1] is the recall level, $r_{k}$ is the recall value at the k-th operating point (threshold), and $p_{\mathrm{interp}}(r_{k})$ is the interpolated precision at recall $r_{k}$ (commonly defined as the maximum precision obtained for any recall $\tilde{r}\geq r_{k}$). The summation form is a discrete approximation of the integral using sampled points on the precision–recall curve.

Computational complexity is reported as GFLOPs, defined as the total number of floating-point operations for a single forward pass divided by $10^{9}$ (counting one multiply–accumulate as two operations). For a standard convolution layer, FLOPs are computed as Equation (3).

(3) $$\begin{array}{cccc} & FLOPs=2\cdot H_{\mathrm{out}}W_{\mathrm{out}}C_{\mathrm{out}}\left(\frac{C_{\mathrm{in}}}{g}\right)K_{h}K_{w}, & & \end{array}$$

In Equation (3), $H_{\mathrm{out}}$ and $W_{\mathrm{out}}$ are the output feature map height and width, $C_{\mathrm{out}}$ is the number of output channels, $C_{\mathrm{in}}$ is the number of input channels, g is the number of groups in grouped convolution (with g=1 for standard convolution), and $K_{h}$ and $K_{w}$ are the kernel height and width, respectively. The factor 2 accounts for one multiplication and one addition per multiply–accumulate operation. The network GFLOPs is obtained by summing FLOPs across all layers and normalizing by $10^{9}$, as shown in Equation (4) where ${FLOPs}_{l}$ denotes the FLOPs of the l-th layer in the model.

(4) $$\begin{array}{c} GFLOPs=\frac{\sum_{l}{FLOPs}_{l}}{10^{9}}, \end{array}$$

Table 2 compares the baseline and StaticPigDetv2 across YOLOv8/10/12 (n/s/m/l) in the unseen Hadong pig pen test set. StaticPigDetv2 improves AP_50_ and F1-Score for all variants, with gains typically driven by higher recall (fewer missed pigs). For example, YOLOv12m increases Precision/Recall/AP_50_/F1-Score from 0.767/0.696/0.758/0.730 (baseline) to 0.883/0.792/0.860/0.835 (proposed), corresponding to +0.116 Precision, +0.096 Recall, +0.102 AP_50_, and +0.105 F1-Score. Similarly, YOLOv8s improves AP_50_ from 0.740 to 0.821 and F1-Score from 0.715 to 0.808, while YOLOv10m improves AP_50_ from 0.739 to 0.834 and F1-Score from 0.710 to 0.803. Although a few cases show small precision drops (e.g., YOLOv12n: 0.824 to 0.796; YOLOv10l: 0.839 to 0.812), recall increases substantially in these models (YOLOv12n: 0.620 to 0.704; YOLOv10l: 0.630 to 0.742), which still yields higher AP_50_ and F1-Score. Overall, the results suggest that combining background-suppressed cues (BIG), facility masking (FIG), and dual-stream fusion (BSI/BIM) is especially effective at recovering pigs under occlusion and in visually novel pen layouts.

Table 3 reports detection accuracy (AP50, consistent with Table 2) together with computational cost (GFLOPs) and runtime performance (latency) measured on an RTX 3060 GPU and the embedded Jetson Orin Nano board. Although the proposed method requires additional time to generate the background-suppressed image, this step is lightweight, adding less than 0.01 ms of latency. In addition, the background image and facility mask are generated once per static test environment and are therefore excluded from the per-image latency measurement.

For the medium and large variants, StaticPigDetv2 improves both accuracy and latency. For example, YOLOv12m increases AP_50_ from 0.758 to 0.860 while reducing GFLOPs from 33.90 to 9.20 and latency from 18.48 ms to 9.18 ms on the RTX 3060; on the Jetson Orin Nano, latency decreases from 67.18 ms to 41.14 ms. Similarly, YOLOv8l increases AP_50_ from 0.799 to 0.848 while reducing latency from 24.34 ms to 10.31 ms on the RTX 3060 and from 74.20 ms to 41.51 ms on the Jetson Orin Nano.

The computational reduction mainly stems from the modified first-stage design in BSI, which applies stride-2 downsampling earlier. As illustrated in Figure 5, this reduces the spatial resolution entering later stages and yields substantial GFLOPs reductions across all model sizes (e.g., YOLOv8m: 39.50 to 10.40 GFLOPs; YOLOv10l: 63.60 to 16.60 GFLOPs; YOLOv12l: 44.70 to 11.90 GFLOPs). Because most backbone and neck layers subsequently operate on smaller feature maps, the proposed models typically achieve lower latency despite the additional dual-input fusion layers.

In contrast, the nano variants exhibit a small runtime regression because their baseline backbones are already highly lightweight, making the fixed overhead of the added fusion layers relatively more pronounced. This leads to reduced GFLOPs but slightly increased latency. For instance, YOLOv8n reduces GFLOPs from 4.1 to 1.2 while latency increases from 4.03 ms to 4.59 ms on the RTX 3060 and from 10.56 ms to 11.13 ms on the Jetson Orin Nano. Similarly, YOLOv10n reduces GFLOPs from 4.2 to 1.3 while latency increases from 4.87 ms to 5.35 ms on the RTX 3060 and from 19.36 ms to 20.27 ms on the Jetson Orin Nano. YOLOv12n reduces GFLOPs from 3.2 to 1.0 while latency increases from 7.90 ms to 8.42 ms on the RTX 3060 and from 27.45 ms to 29.42 ms on the Jetson Orin Nano. Nevertheless, these nano models still achieve AP_50_ gains (e.g., YOLOv8n: 0.717 to 0.788), suggesting that StaticPigDetv2 remains beneficial when deployment constraints favor smaller detectors.

Experiments evaluating the effect of applying facility masks generated by FIG to the input images are summarized in Table 4. In both configurations, the detector uses the background-suppressed images produced by BIG and the 3D convolution-based feature fusion of BSI; the only difference is whether the facility mask is applied to the inputs.

Applying the facility mask yields higher AP_50_ and F1-Score for most models. The AP_50_ improvement ranges from approximately 1% to 10%, with the largest gain observed for the YOLOv12m model, and the F1-Score improvement ranges from approximately 3% to 10%, again with the largest improvement for YOLOv12m. Although a few model variants exhibit small decreases in AP_50_ or F1-Score, the highest recorded accuracy is obtained with the facility-masked YOLOv12m model, and most models show clear accuracy gains. These results indicate that masking facility regions during training helps the detector better detect objects in unseen environments.

Table 5 studies the SoftMax multiplier used to scale the background-suppressed feature branch inside BSI. A 2× multiplier provides the most consistent performance across models: for YOLOv12m, AP_50_ increases from 0.828 (1×) to 0.860 (2×), with F1-Score improving from 0.786 to 0.835. While some models prefer stronger scaling (e.g., YOLOv12n AP_50_: 0.745/0.748/0.768 for 1×/2×/3×), the 2× setting offers a stable trade-off that avoids the occasional degradation observed at 3× (e.g., YOLOv8n AP_50_ drops to 0.740 at 3×).

Table 6 compares three fusion strategies for combining features from the original and background-suppressed streams: element-wise addition, concatenation followed by a 2D convolution, and the proposed 3D convolution. The 3D convolution consistently yields the highest AP_50_ and F1-Score across all backbones. For example, on YOLOv10m, AP_50_ improves from 0.806 (addition) and 0.816 (concat + 2DConv) to 0.834 (3DConv), and F1-Score improves from 0.762 and 0.772 to 0.803. On YOLOv8n, AP_50_ increases from 0.766/0.764 to 0.788 with 3DConv. Although recall can be slightly higher with alternative fusions in isolated cases (e.g., YOLOv8s recall 0.771 with concat + 2DConv vs. 0.754 with 3DConv), 3DConv better preserves the overall precision–recall balance, leading to higher AP_50_/F1-Score.

Table 7 compares a standard difference-image input with the proposed background-suppressed image produced by BIG. The background-suppressed representation is generally more effective because it preserves the original foreground texture while zeroing out background pixels. This yields notable gains for mid-to-large models (e.g., YOLOv8m AP_50_: 0.771 to 0.806; F1-Score: 0.741 to 0.777, and YOLOv12l AP_50_: 0.797 to 0.838; F1-Score: 0.791 to 0.816). For some small models, the difference image can be competitive (YOLOv8n F1-Score: 0.790 vs. 0.781), likely because the residual-only representation simplifies the input. Overall, BIG’s background-suppressed image provides a richer and more transferable signal for unseen environments than raw pixel residuals.

Table 8 compares the proposed method to DOG [17], which also targets unseen-environment robustness using depth-oriented preprocessing. Across all model variants, StaticPigDetv2 achieves higher accuracy, especially by improving recall. For example, on YOLOv12m, DOG obtains Precision/Recall/AP_50_/F1-Score of 0.790/0.649/0.706/0.713, whereas StaticPigDetv2 reaches 0.883/0.792/0.860/0.835 with increase of 0.154 on AP_50_ and 0.122 on F1-Score. On YOLOv10l, AP_50_ increases from 0.699 (DOG) to 0.791 (proposed) and F1-Score from 0.669 to 0.775. These results indicate that explicitly modeling both background suppression (BIG) and facility occlusions (FIG), together with dual-stream fusion (BSI), provides additional generalization benefits beyond depth-oriented grayscale preprocessing alone.

To assess robustness across unseen environment, Table 9 reports results on the secondary Jochiwon dataset. StaticPigDetv2 improves F1-Score for all evaluated models, with especially large gains for YOLOv10s (F1-Score: 0.652 to 0.807; AP_50_: 0.681 to 0.808). Several variants also show clear precision improvements (e.g., YOLOv8n Precision: 0.787 to 0.897), while recall changes are generally modest. In a few cases, AP_50_ decreases despite an F1-Score increase (e.g., YOLOv12m AP_50_: 0.772 to 0.749; F1-Score: 0.735 to 0.756), suggesting that the proposed preprocessing and fusion mainly stabilizes correct detections at the operating point used for F1-Score, while the IoU distribution across matched boxes can vary by scene. Overall, the consistent F1-Score improvements across two distinct unseen farms support the claim that StaticPigDetv2 mitigates domain gaps without environment-specific retraining.

**Figure 7:**
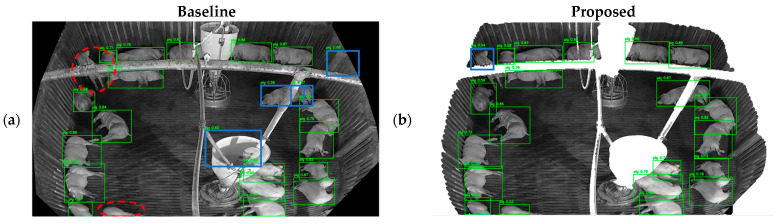

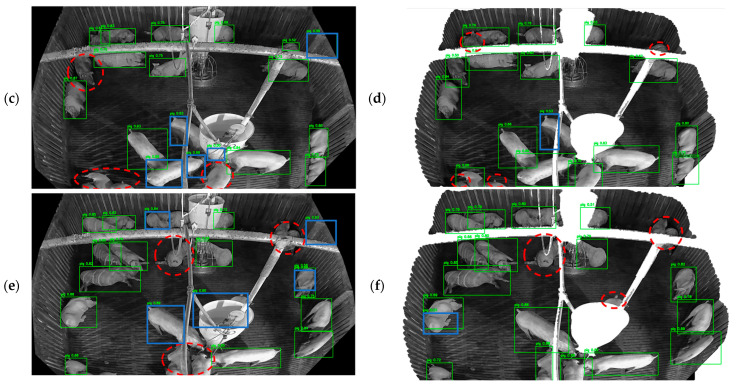
Visual representation of Hadong detection results comparing the baseline and proposed StaticPigDetv2 methods. Green boxes show correctly detected cases. False negative errors are marked with dotted circle. False positive errors are marked with solid blue rectangle.

Figure 7 illustrates the differences in Hadong detection results between the baseline and the proposed method. Each image pair corresponds to the same scene, with detections obtained from models trained under the two different settings. The YOLOv12m model, which achieved the highest accuracy among all evaluated models, was used for this comparison. False positives are marked with blue rectangles while false negatives are marked with dotted red circles. In Figure 7a, detection boxes that are fragmented by facility pipes are reconnected in Figure 7b, and missed detections due to occlusion by the feeding facility and partially visible pigs lying near the bottom of the image are recovered. However, pigs that are occluded by the pipe located around upper-left corner in Figure 7a are partially detected in Figure 7b. Similarly, in Figure 7c, pigs occluded by facility pipes and those located at the bottom of the image are not detected, whereas in Figure 7d these pigs are mostly correctly identified. The false positives associated with the large feeding facility disappear. Although a few pigs remain undetected, many false negative and false positive detection boxes by the facility pipe in Figure 7e are resolved in Figure 7f. Additionally, false positive detection boxes by the upper right corner in all three examples disappear with the proposed StaticPigDetv2 method. Overall, Figure 7 demonstrates that while some errors occur in proposed method, many detection errors arising in unseen environments are mitigated by the proposed method.

**Figure 8:**
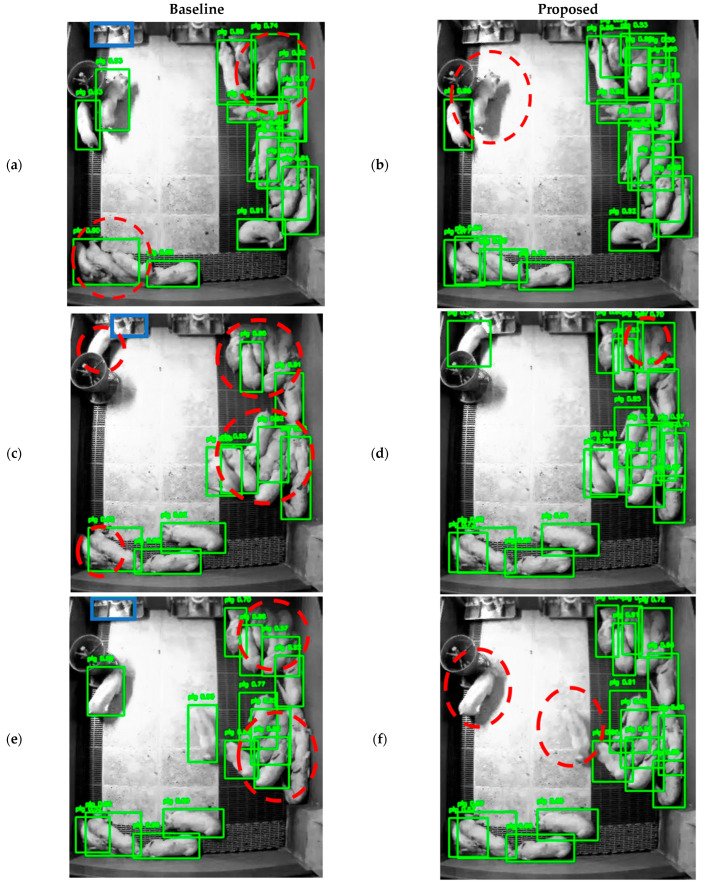
Visual representation of Jochiwon detection results comparing the baseline and proposed StaticPigDetv2 methods. Green boxes show correctly detected cases. False negative errors are marked with a dotted circle. False positive errors are marked with solid blue rectangle.

Figure 8 illustrates the differences in Jochiwon detection results between the baseline and the proposed method. Each image pair corresponds to the same scene, with detections obtained from models trained under two different settings. Unlike the Hadong dataset, the Jochiwon dataset does not have any occluding facility, hence, there is no facility mask. The YOLOv12m model was used for this comparison. False positives are marked with blue rectangles, while false negatives are marked with dotted red circles. In the three examples shown in Figure 8a,c,e, false positives caused by the feeding facility are effectively mitigated in the corresponding proposed method’s results (Figure 8b,d,f). In Figure 8a, detection boxes that are merged into a single box for multiple clustered pigs are mostly separated into individual detections in Figure 8b. However, a pig located on a white surface in Figure 8a is missed in Figure 8b. Similarly, in Figure 8c, detection boxes that are grouped into one box for multiple pigs on the right side are largely separated in Figure 8d, although a few false negatives remain. In addition, missed detections in the upper-left and bottom-left corners in Figure 8c are correctly detected in Figure 8d. Lastly, while multiple pigs that are undetected in Figure 8e are mostly detected in Figure 8f, two pigs near the white surface are missed. Overall, Figure 8 demonstrates that although some errors still occur with the proposed method, many detection failures observed in different unseen environments are alleviated.

## 5. Conclusions

Unseen pig monitoring environments pose a substantial challenge for accurate pig detection. When there are discrepancies between the training and test datasets in terms of background or facility appearance, numerous detection errors occur despite recent advances in object detectors.

In this study, we proposed a method to improve both detection accuracy and latency by using BIG, FIG, and BSI, which explicitly leverage background and facility information. BIG (Background-suppressed Image Generator) produces background-suppressed images that reduce the impact of background information during training. FIG (Facility-mask Image Generator) generates facility masks that localize facility regions and enhance the features of pig objects occluded by these facilities. BSI (Background-Suppressed Integration) integrates background-suppressed information into the original feature maps to create a leverage-like feature map.

The proposed method improved detection performance in unseen pig monitoring environments across various YOLO models, including YOLOv8, YOLOv10, and YOLOv12. The proposed method improved the baseline model’s performance with YOLOv8 (accuracy from 79.9% to 84.8% and latency from 74.20 ms to 41.51 ms), YOLOv10 (accuracy from 73.9% to 83.4% and latency from 49.85 ms to 32.47 ms), and YOLOv12 (accuracy from 75.8% to 86.0% and latency from 67.18 ms to 41.14 ms). Future work will explore additional feature fusion strategies to further improve feature representation using background information. Also, criteria for defining nano/small/medium/large models for StaticPigDetv2 will be studied in order to consistently provide better accuracy with bigger models. Additionally, future work will also consider quantitative evaluation of the depth estimation and/or inpainting components on pig farm datasets (e.g., assessing depth prediction fidelity and inpainting quality under farm-specific occlusions), to better characterize how the quality of these intermediate outputs relates to downstream detection performance.

## Figures and Tables

**Table 1:** Some of the recent results for group-housed pig detection (published during 2021–2025). Most of the previous results have focused on seen environment (e.g., train/test with the same dataset) and improved accuracy only (i.e., even better than 99% accuracy reported). To the best of our knowledge, none of the recent results could improve both accuracy and latency, especially for unseen environment. The proposed method is aimed to improve both the accuracy and latency by exploiting depth-based background and facility information.

Reference	Target Environment (Seen vs. Unseen)	Target Performance (Accuracy vs. Latency vs. Both)	Details
[31]	Seen (train/test: seen Chinese Pigs)	Accuracy (92.4%)	Establishes a benchmark dataset and evaluates standard CNNs for classifying standing, sitting, and lying postures.
[40]	Seen (train/test: seen Korean Pigs)	Accuracy (79.9%->94.3%)	Combines multiple deep learning models into an ensemble to improve detection precision in crowded commercial pens.
[15]	Seen (train/test: seen Chinese Pig)	Accuracy (36.6%->90.3%)	Improves tracking consistency by combining Joint Probability Data Association with particle filters to handle occlusions.
[49]	Seen (train/test: seen Chinese Pig)	Accuracy (93.1%)	Utilizes spatial and channel attention within a Feature Pyramid Network to detect individual pigs in group housing.
[54]	Seen (train/test: seen Korean Pig)	Accuracy (98.6%)	Monitors behavioral changes (standing vs. lying) in response to varying greenhouse gas levels in experimental barns.
[41]	Seen (train/test: seen German Pig)	Accuracy (95.1%)	Quantifies social contact frequency by detect pig movements and detecting proximity between specific body parts.
[52]	Seen (train/test: seen Chinese Pig)	Accuracy (96.0%)	Developed for multi-part detection (head, tail, body) to monitor health indicators like tail-biting in intensive farming.
[53]	Seen (train/test: seen multiple dataset)	Accuracy (99.4%) Speed (100 FPS)	Separates detection and classification into a two-stage pipeline to improve posture recognition accuracy without sacrificing speed.
[55]	Seen (train/test: seen Chinese Pig)	Accuracy (96.8%)	Focuses on non-contact monitoring of large-frame sow behavior to identify estrus through vulvar image analysis.
[56]	Seen (train/test: seen Chinese Pig)	Accuracy (93.6%) Latency (27 ms)	Modifies the CenterNet architecture to provide a faster and more accurate anchor-free approach for pig face identification.
[16]	Seen (train/test: seen Korean Pig)	Accuracy (99.4%) Latency (25.8 ms)	Optimized for the Jetson Nano to count pigs passing through hallways using a lightweight detection and tracking algorithm.
[44]	Seen (train/test: seen Chinese Pig)	Accuracy (88.3%->95.3%) Speed (5.8 FPS->6.3 FPS)	Uses Graph Convolutional Networks to model social interactions between piglets based on their physical spatial relationships.
[19]	Seen (train/test: seen Korean Pigs)	Accuracy (76.60%->90.6%) Latency (4.8 ms)	Uses a Generative Adversarial Network to remove “haze” from IR camera lenses, significantly boosting detection in dirty farm environments.
[57]	Seen (train/test: seen Chinese Pig)	Accuracy (99.5%) Latency (9.5 ms)	Implements an improved YOLOX model to simultaneously detect pig position and complex postures like sitting.
[21]	Seen (train/test: seen Korean Pigs)	Accuracy (84%->94%)	Enhances detection by using background subtraction and facility information to filter out false positives from pen structures.
[51]	Seen (train/test: seen multiple dataset)	Accuracy (99.5%)	Introduces a semi-shuffling data method to prevent training–test overlap when identifying social interactions.
[45]	Seen (train/test: seen Chinese Pig)	Accuracy (87%->95%)	Utilizes a spatial–temporal backbone (TransNeXt) to recognize feeding behaviors and identify dangerous health anomalies.
[32]	Seen (train/test: seen multiple dataset)	Accuracy (96%)	Incorporates a mask-scoring branch to ensure segment quality, improving posture detection in highly overlapping groups.
[43]	Seen (train/test: seen Chinese Pig)	Accuracy (98.7%)	Established a pig behavior detection model based on an improved Mask R-CNN incorporating the CBAM attention mechanism.
[39]	Seen (Not specified)	Accuracy (91.0%->93.7%)	Integrates Multi-Head Self-Attention and Depthwise Separable convolutions for efficient, real-time counting.
[47]	Seen (train/test: seen Chinese Pig)	Accuracy (85.1%->91.9%)	Uses multiscale feature enhancement to detect pigs under complex lighting and high-density conditions.
[46]	Seen (train/test: seen Scotland dataset)	Accuracy (61.7%->75.9%)	Proposes a spatial-temporal perception network to model 13 different welfare-related behaviors in videos.
[17]	Unseen (train: German Pigs, test: unseen Pigs)	Accuracy (93%->97%)	Uses depth information to create high-contrast gray images that allow models to work in unseen farms without retraining. Tests with Top-View images.
[18]	Seen (train/test: seen Korean Pigs)	Accuracy (36%->90%)	Solves domain shift using only one labeled target image (SLOT) plus augmentation search + self-training, boosting AP dramatically under domain shift.
**Proposed Method**	**Unseen** **(train: German Pigs,** **test: unseen Korean Pigs)**	**Accuracy** **(75%->86%)** **and Latency** **(67 ms** **-** **>41 ms)**	**Modifies YOLOv8, YOLOv10, YOLOv12** **.** **Modifies input images by using** **generated background image.** **Tests with both Tilted and Top-View images.**

**Table 2:** Accuracy performance of different YOLO model versions tested to compare the baseline and proposed StaticPigDetv2 methods. Proposed method is marked with bold. The symbol Δ represents the difference from proposed method to baseline.

Detection Model	Method	Precision ↑	Recall ↑	AP_50_ ↑	F1-Score ↑
YOLOv8n	Baseline	0.727	0.650	0.717	0.686
	**Proposed**	**0.834**	**0.735**	**0.788**	**0.781**
	Δ	+0.107	+0.085	+0.071	+0.095
YOLOv8s	Baseline	0.759	0.675	0.740	0.715
	**Proposed**	**0.87** **0**	**0.754**	**0.821**	**0.808**
	Δ	+0.111	+0.079	+0.081	+0.093
YOLOv8m	Baseline	0.822	0.684	0.781	0.747
	**Proposed**	**0.834**	**0.728**	**0.806**	**0.777**
	Δ	+0.012	+0.044	+0.025	+0.031
YOLOv8l	Baseline	0.862	0.642	0.799	0.736
	**Proposed**	**0.898**	**0.76** **0**	**0.848**	**0.823**
	Δ	+0.036	+0.118	+0.049	+0.087
YOLOv10n	Baseline	0.760	0.601	0.692	0.671
	**Proposed**	**0.848**	**0.684**	**0.744**	**0.757**
	Δ	+0.088	+0.083	+0.052	+0.086
YOLOv10s	Baseline	0.788	0.640	0.732	0.706
	**Proposed**	**0.841**	**0.735**	**0.783**	**0.784**
	Δ	+0.053	+0.095	+0.051	+0.078
YOLOv10m	Baseline	0.762	0.665	0.739	0.710
	**Proposed**	**0.855**	**0.757**	**0.834**	**0.803**
	Δ	+0.093	+0.092	+0.095	+0.093
YOLOv10l	Baseline	0.839	0.630	0.730	0.720
	**Proposed**	**0.812**	**0.742**	**0.791**	**0.775**
	Δ	−0.027	+0.112	+0.061	+0.056
YOLOv12n	Baseline	0.824	0.620	0.702	0.708
	**Proposed**	**0.796**	**0.704**	**0.748**	**0.747**
	Δ	−0.028	+0.084	+0.046	+0.040
YOLOv12s	Baseline	0.778	0.646	0.746	0.706
	**Proposed**	**0.878**	**0.741**	**0.813**	**0.804**
	Δ	+0.100	+0.095	+0.067	+0.098
YOLOv12m	Baseline	0.767	0.696	0.758	0.730
	**Proposed**	**0.883**	**0.792**	**0.86** **0**	**0.835**
	Δ	+0.116	+0.096	+0.102	+0.105
YOLOv12l	Baseline	0.864	0.672	0.780	0.756
	**Proposed**	**0.866**	**0.771**	**0.838**	**0.816**
	Δ	+0.002	+0.099	+0.058	+0.060

**Table 3:** Accuracy and latency related performance of different YOLO model versions tested to compare the baseline and proposed StaticPigDetv2 methods. Proposed method is marked with bold. The symbol Δ represents the difference from proposed method to baseline.

Detection Model	Method	AP_50_ ↑	GFLOPs ↓	Latency ↓ (ms) RTX 3060	Latency ↓ (ms) Jetson Orin Nano
YOLOv8n	Baseline	0.717	4.1	4.03	10.56
	**Proposed**	**0.788**	**1.2**	**4.** **59**	**11.13**
	Δ	+0.071	−2.9	+0.56	+0.57
YOLOv8s	Baseline	0.740	14.3	6.93	19.76
	**Proposed**	**0.821**	**3.9**	**4.** **63**	**13.25**
	Δ	+0.081	−10.4	−2.30	−6.51
YOLOv8m	Baseline	0.781	39.5	16.78	44.93
	**Proposed**	**0.806**	**10.4**	**7.37**	**33.63**
	Δ	+0.025	−29.1	-9.41	−11.30
YOLOv8l	Baseline	0.799	82.7	24.34	74.20
	**Proposed**	**0.848**	**21.4**	**10.31**	**41.51**
	Δ	+0.049	−61.3	−14.03	−32.69
YOLOv10n	Baseline	0.692	4.2	4.87	19.36
	**Proposed**	**0.744**	**1.3**	**5.35**	**20.27**
	Δ	+0.052	−2.9	+0.48	+0.91
YOLOv10s	Baseline	0.732	12.4	6.72	28.05
	**Proposed**	**0.783**	**3.5**	**5.45**	**22.75**
	Δ	+0.051	−8.9	−1.27	−5.30
YOLOv10m	Baseline	0.739	32.0	14.64	49.85
	**Proposed**	**0.834**	**8.6**	**6.78**	**32.47**
	Δ	+0.095	−23.4	−7.86	−17.38
YOLOv10l	Baseline	0.730	63.6	21.62	80.28
	**Proposed**	**0.791**	**16.6**	**8.74**	**39.48**
	Δ	+0.061	−47.0	−12.88	−40.80
YOLOv12n	Baseline	0.702	3.2	7.90	27.45
	**Proposed**	**0.748**	**1** **.0**	**8.42**	**29.42**
	Δ	+0.046	−2.2	+0.52	+1.97
YOLOv12s	Baseline	0.746	10.8	9.39	34.56
	**Proposed**	**0.813**	**3** **.0**	**8.60**	**30.57**
	Δ	+0.067	−7.8	−0.79	−3.99
YOLOv12m	Baseline	0.758	33.9	18.48	67.18
	**Proposed**	**0.86** **0**	**9.2**	**9.18**	**41.14**
	Δ	+0.102	−24.7	−9.30	−26.04
YOLOv12l	Baseline	0.780	44.7	27.32	104.28
	**Proposed**	**0.838**	**11.9**	**14.35**	**68.52**
	Δ	+0.058	−32.8	−12.97	−35.76

**Table 4:** Accuracy performance of different YOLO model versions tested to evaluate the effectiveness of FIG in the proposed StaticPigDetv2 method. Usage of FIG is marked with bold. The symbol Δ represents the difference from the usages of FIG to baseline.

Detection Model	FIG	Precision ↑	Recall ↑	AP_50_ ↑	F1-Score ↑
YOLOv8n	X	0.811	0.674	0.748	0.736
	Δ	+0.084	+0.024	+0.031	+0.050
	**O**	**0.834**	**0.735**	**0.788**	**0.781**
	Δ	+0.107	+0.085	+0.071	+0.095
YOLOv8s	X	0.861	0.719	0.803	0.784
	Δ	+0.102	+0.044	+0.063	+0.069
	**O**	**0.87** **0**	**0.754**	**0.821**	**0.808**
	Δ	+0.111	+0.079	+0.081	+0.093
YOLOv8m	X	0.879	0.734	0.842	0.800
	Δ	+0.057	+0.050	+0.061	+0.053
	**O**	**0.834**	**0.728**	**0.806**	**0.777**
	Δ	+0.012	+0.044	+0.025	+0.030
YOLOv8l	X	0.839	0.762	0.832	0.799
	Δ	−0.023	+0.120	+0.033	+0.063
	**O**	**0.898**	**0.76** **0**	**0.848**	**0.823**
	Δ	+0.036	+0.118	+0.049	+0.087
YOLOv10n	X	0.829	0.664	0.734	0.737
	Δ	+0.069	+0.063	+0.042	+0.066
	**O**	**0.848**	**0.684**	**0.744**	**0.757**
	Δ	+0.088	+0.083	+0.052	+0.086
YOLOv10s	X	0.848	0.730	0.804	0.785
	Δ	+0.060	+0.090	+0.072	+0.079
	**O**	**0.841**	**0.735**	**0.783**	**0.784**
	Δ	+0.053	+0.095	+0.051	+0.078
YOLOv10m	X	0.894	0.713	0.808	0.793
	Δ	+0.132	+0.048	+0.069	+0.083
	**O**	**0.855**	**0.757**	**0.834**	**0.803**
	Δ	+0.093	+0.092	+0.095	+0.093
YOLOv10l	X	0.854	0.702	0.790	0.771
	Δ	+0.015	+0.072	+0.060	+0.051
	**O**	**0.812**	**0.742**	**0.791**	**0.775**
	Δ	−0.027	+0.112	+0.061	+0.055
YOLOv12n	X	0.809	0.645	0.720	0.718
	Δ	−0.015	+0.025	+0.018	+0.010
	**O**	**0.796**	**0.704**	**0.748**	**0.747**
	Δ	−0.028	+0.084	+0.046	+0.039
YOLOv12s	X	0.855	0.709	0.780	0.775
	Δ	+0.077	+0.063	+0.034	+0.069
	**O**	**0.878**	**0.741**	**0.813**	**0.804**
	Δ	+0.100	+0.095	+0.067	+0.098
YOLOv12m	X	0.861	0.743	0.833	0.798
	Δ	+0.094	+0.047	+0.075	+0.068
	**O**	**0.883**	**0.792**	**0.86** **0**	**0.835**
	Δ	+0.116	+0.096	+0.102	+0.105
YOLOv12l	X	0.841	0.750	0.820	0.793
	Δ	−0.023	+0.078	+0.040	+0.037
	**O**	**0.866**	**0.771**	**0.838**	**0.816**
	Δ	+0.002	+0.099	+0.058	+0.060

**Table 5:** Accuracy performance of different YOLO model versions tested to compare different SoftMax multipliers applied in BSI. Multiplier that is used in proposed method is marked with bold.

Detection Model	SoftMax Multiplier	Precision ↑	Recall ↑	AP_50_ ↑	F1-Score ↑
YOLOv8n	1×	0.833	0.727	0.774	0.776
	**2×**	**0.834**	**0.735**	**0.788**	**0.781**
	3×	0.834	0.676	0.740	0.747
YOLOv8s	1×	0.860	0.737	0.808	0.794
	**2×**	**0.870**	**0.754**	**0.821**	**0.808**
	3×	0.852	0.761	0.830	0.804
YOLOv8m	1×	0.850	0.739	0.820	0.791
	**2×**	**0.834**	**0.728**	**0.806**	**0.777**
	3×	0.826	0.766	0.822	0.795
YOLOv8l	1×	0.858	0.758	0.831	0.805
	**2×**	**0.898**	**0.760**	**0.848**	**0.823**
	3×	0.854	0.771	0.831	0.810
YOLOv10n	1×	0.802	0.661	0.723	0.725
	**2×**	**0.848**	**0.684**	**0.744**	**0.757**
	3×	0.853	0.682	0.754	0.758
YOLOv10s	1×	0.846	0.716	0.783	0.776
	**2×**	**0.841**	**0.735**	**0.783**	**0.784**
	3×	0.854	0.714	0.778	0.778
YOLOv10m	1×	0.849	0.773	0.824	0.809
	**2×**	**0.855**	**0.757**	**0.834**	**0.803**
	3×	0.834	0.753	0.822	0.791
YOLOv10l	1×	0.859	0.733	0.827	0.791
	**2×**	**0.812**	**0.742**	**0.791**	**0.775**
	3×	0.825	0.727	0.791	0.773
YOLOv12n	1×	0.792	0.695	0.745	0.740
	**2×**	**0.796**	**0.704**	**0.748**	**0.747**
	3×	0.804	0.723	0.768	0.761
YOLOv12s	1×	0.854	0.740	0.799	0.793
	**2×**	**0.878**	**0.741**	**0.813**	**0.804**
	3×	0.841	0.776	0.827	0.807
YOLOv12m	1×	0.831	0.746	0.828	0.786
	**2×**	**0.883**	**0.792**	**0.860**	**0.835**
	3×	0.871	0.753	0.842	0.808
YOLOv12l	1×	0.888	0.760	0.841	0.794
	**2×**	**0.866**	**0.771**	**0.838**	**0.816**
	3×	0.864	0.781	0.842	0.824

**Table 6:** Accuracy performance of different YOLO model versions tested to compare different feature fusion methods which are addition, combination of concatenation and 2d Convolution, and 3D Convolution. Fusion method that is used in the proposed method is marked with bold.

Detection Model	Fusion Methods	Precision ↑	Recall ↑	AP_50_ ↑	F1-Score ↑
YOLOv8n	Addition	0.818	0.699	0.766	0.754
	Concat + 2DConv	0.807	0.688	0.764	0.743
	**3DConv**	**0.834**	**0.735**	**0.788**	**0.781**
YOLOv8s	Addition	0.835	0.737	0.819	0.783
	Concat + 2DConv	0.834	0.771	0.806	0.801
	**3DConv**	**0.870**	**0.754**	**0.821**	**0.808**
YOLOv8m	Addition	0.788	0.698	0.783	0.740
	Concat + 2DConv	0.770	0.742	0.784	0.756
	**3DConv**	**0.834**	**0.728**	**0.806**	**0.777**
YOLOv8l	Addition	0.837	0.753	0.833	0.793
	Concat + 2DConv	0.883	0.739	0.826	0.805
	**3DConv**	**0.898**	**0.760**	**0.848**	**0.823**
YOLOv10n	Addition	0.749	0.680	0.735	0.713
	Concat + 2DConv	0.802	0.675	0.758	0.733
	**3DConv**	**0.848**	**0.684**	**0.744**	**0.757**
YOLOv10s	Addition	0.791	0.678	0.760	0.730
	Concat + 2DConv	0.810	0.718	0.778	0.761
	**3DConv**	**0.841**	**0.735**	**0.783**	**0.784**
YOLOv10m	Addition	0.796	0.730	0.806	0.762
	Concat + 2DConv	0.797	0.748	0.816	0.772
	**3DConv**	**0.855**	**0.757**	**0.834**	**0.803**
YOLOv10l	Addition	0.797	0.743	0.781	0.769
	Concat + 2DConv	0.792	0.698	0.782	0.742
	**3DConv**	**0.812**	**0.742**	**0.791**	**0.775**
YOLOv12n	Addition	0.790	0.672	0.725	0.726
	Concat + 2DConv	0.805	0.665	0.718	0.728
	**3DConv**	**0.796**	**0.704**	**0.748**	**0.747**
YOLOv12s	Addition	0.751	0.757	0.799	0.754
	Concat + 2DConv	0.767	0.756	0.791	0.762
	**3DConv**	**0.878**	**0.741**	**0.813**	**0.804**
YOLOv12m	Addition	0.854	0.773	0.852	0.811
	Concat + 2DConv	0.864	0.769	0.840	0.814
	**3DConv**	**0.883**	**0.792**	**0.860**	**0.835**
YOLOv12l	Addition	0.852	0.759	0.838	0.803
	Concat + 2DConv	0.807	0.791	0.829	0.799
	**3DConv**	**0.866**	**0.771**	**0.838**	**0.816**

**Table 7:** Accuracy performance of different YOLO model versions tested to compare the difference image and proposed background-suppressed image used in BIG module. Proposed method is marked with bold.

Detection Model	Method	Precision ↑	Recall ↑	AP_50_ ↑	F1-Score ↑
YOLOv8n	Diff Image	0.839	0.746	0.785	0.790
	**Proposed**	**0.834**	**0.735**	**0.788**	**0.781**
YOLOv8s	Diff Image	0.834	0.774	0.804	0.803
	**Proposed**	**0.870**	**0.754**	**0.821**	**0.808**
YOLOv8m	Diff Image	0.740	0.743	0.771	0.741
	**Proposed**	**0.834**	**0.728**	**0.806**	**0.777**
YOLOv8l	Diff Image	0.865	0.737	0.827	0.796
	**Proposed**	**0.898**	**0.760**	**0.848**	**0.823**
YOLOv10n	Diff Image	0.819	0.650	0.722	0.725
	**Proposed**	**0.848**	**0.684**	**0.744**	**0.757**
YOLOv10s	Diff Image	0.841	0.693	0.780	0.760
	**Proposed**	**0.841**	**0.735**	**0.783**	**0.784**
YOLOv10m	Diff Image	0.827	0.765	0.819	0.795
	**Proposed**	**0.855**	**0.757**	**0.834**	**0.803**
YOLOv10l	Diff Image	0.776	0.737	0.775	0.756
	**Proposed**	**0.812**	**0.742**	**0.791**	**0.775**
YOLOv12n	Diff Image	0.794	0.694	0.732	0.741
	**Proposed**	**0.796**	**0.704**	**0.748**	**0.747**
YOLOv12s	Diff Image	0.850	0.735	0.791	0.788
	**Proposed**	**0.878**	**0.741**	**0.813**	**0.804**
YOLOv12m	Diff Image	0.870	0.777	0.830	0.821
	**Proposed**	**0.883**	**0.792**	**0.860**	**0.835**
YOLOv12l	Diff Image	0.812	0.770	0.797	0.791
	**Proposed**	**0.866**	**0.771**	**0.838**	**0.816**

**Table 8:** Accuracy performance of different YOLO model versions tested to compare the DOG [17] and proposed StaticPigDetv2 methods. Proposed method is marked with bold.

Detection Model	Method	Precision ↑	Recall ↑	AP_50_ ↑	F1-Score ↑
YOLOv8n	DOG	0.772	0.621	0.711	0.688
	**Proposed**	**0.834**	**0.735**	**0.788**	**0.781**
YOLOv8s	DOG	0.802	0.686	0.751	0.739
	**Proposed**	**0.870**	**0.754**	**0.821**	**0.808**
YOLOv8m	DOG	0.812	0.687	0.772	0.744
	**Proposed**	**0.834**	**0.728**	**0.806**	**0.777**
YOLOv8l	DOG	0.773	0.659	0.737	0.711
	**Proposed**	**0.898**	**0.760**	**0.848**	**0.823**
YOLOv10n	DOG	0.768	0.558	0.650	0.646
	**Proposed**	**0.848**	**0.684**	**0.744**	**0.757**
YOLOv10s	DOG	0.799	0.658	0.740	0.722
	**Proposed**	**0.841**	**0.735**	**0.783**	**0.784**
YOLOv10m	DOG	0.759	0.643	0.727	0.696
	**Proposed**	**0.855**	**0.757**	**0.834**	**0.803**
YOLOv10l	DOG	0.766	0.594	0.699	0.669
	Proposed	0.812	0.742	0.791	0.775
YOLOv12n	DOG	0.712	0.586	0.618	0.643
	**Proposed**	**0.796**	**0.704**	**0.748**	**0.747**
YOLOv12s	DOG	0.793	0.701	0.756	0.744
	**Proposed**	**0.878**	**0.741**	**0.813**	**0.804**
YOLOv12m	DOG	0.790	0.649	0.706	0.713
	**Proposed**	**0.883**	**0.792**	**0.860**	**0.835**
YOLOv12l	DOG	0.793	0.701	0.765	0.744
	**Proposed**	**0.866**	**0.771**	**0.838**	**0.816**

**Table 9:** Accuracy performance of different YOLO model versions tested to compare the baseline and proposed StaticPigDetv2 methods tested on Jochiwon pig dataset. Proposed method is marked with bold.

Detection Model	Method	Precision ↑	Recall ↑	AP_50_ ↑	F1-Score ↑
YOLOv8n	Baseline	0.787	0.710	0.768	0.747
	**Proposed**	**0.897**	**0.696**	**0.784**	**0.784**
YOLOv8s	Baseline	0.795	0.700	0.792	0.744
	**Proposed**	**0.871**	**0.682**	**0.761**	**0.765**
YOLOv8m	Baseline	0.842	0.685	0.777	0.755
	**Proposed**	**0.887**	**0.719**	**0.783**	**0.794**
YOLOv8l	Baseline	0.851	0.640	0.712	0.731
	**Proposed**	**0.869**	**0.666**	**0.743**	**0.754**
YOLOv10n	Baseline	0.814	0.640	0.712	0.717
	**Proposed**	**0.875**	**0.681**	**0.770**	**0.766**
YOLOv10s	Baseline	0.752	0.575	0.681	0.652
	**Proposed**	**0.903**	**0.729**	**0.808**	**0.807**
YOLOv10m	Baseline	0.813	0.643	0.733	0.718
	**Proposed**	**0.885**	**0.689**	**0.767**	**0.775**
YOLOv10l	Baseline	0.805	0.643	0.722	0.715
	**Proposed**	**0.888**	**0.680**	**0.767**	**0.770**
YOLOv12n	Baseline	0.811	0.616	0.701	0.700
	**Proposed**	**0.884**	**0.694**	**0.769**	**0.778**
YOLOv12s	Baseline	0.826	0.694	0.788	0.754
	**Proposed**	**0.881**	**0.716**	**0.797**	**0.790**
YOLOv12m	Baseline	0.817	0.668	0.772	0.735
	**Proposed**	**0.870**	**0.669**	**0.749**	**0.756**
YOLOv12l	Baseline	0.788	0.709	0.748	0.746
	**Proposed**	**0.848**	**0.691**	**0.767**	**0.761**

## Data Availability

The original contributions presented in this study are included in the article. Further inquiries can be directed to the corresponding author.
